# Dietary cadmium exposure and risk of epithelial ovarian cancer in a prospective cohort of Swedish women

**DOI:** 10.1038/bjc.2011.238

**Published:** 2011-06-21

**Authors:** B Julin, A Wolk, A Åkesson

**Affiliations:** 1 Division of Nutritional Epidemiology, Institute of Environmental Medicine, Karolinska Institutet, Box 210, 171 77 Stockholm, Sweden

**Keywords:** epithelial ovarian cancer, dietary cadmium exposure, oestrogen-mimicking

## Abstract

**Background:**

The proposed cadmium-induced oestrogen mimicking effects in reproductive tissues, suggest a role of this widespread food contaminant in the development of hormone-dependent malignancies.

**Methods:**

We prospectively evaluated the association between tertiles of dietary cadmium exposure and epithelial ovarian cancer in 60 889 women from the population-based Swedish Mammography Cohort. Dietary cadmium was estimated using a food-frequency questionnaire at baseline (1987–1990) and in 1997. Multivariable-adjusted rate ratios (RR) were evaluated using Cox proportional hazards models.

**Results:**

During a mean follow-up of 18.9 years (1 149 470 person-years), we identified 409 incident cases of epithelial ovarian cancer, including 215 serous, 27 mucinous, 62 endometrioid and 12 clear cell tumours. We found no association between dietary cadmium exposure and the risk of ovarian cancer. Compared with the lowest tertile of cadmium exposure, the multivariable-adjusted RR for the highest tertile was 0.90 (95% confidence interval (CI): 0.71–1.15) for total epithelial ovarian cancer. Likewise, no association was observed in subtypes modelled with continuous dietary cadmium exposure; multivariable RR for each 1 *μ*g per day increment of cadmium: 0.97 (95% CI: 0.93–1.02) for serous tumours, 0.94 (95% CI: 0.82–1.07) for mucinous tumours and 1.00 (95% CI: 0.92–1.08) for endometrioid and clear cell tumours.

**Conclusion:**

Our study suggests that dietary cadmium exposure is not likely to have a substantial role in ovarian cancer development.

The food-pollutant cadmium has been widely dispersed into the environment even in industrially non-polluted areas. Farmland and thereby food, may become contaminated by atmospheric deposition and by the use of cadmium-containing fertilizers and sewage sludge (Jarup and Åkesson, 2009). Even though shellfish, offal products and certain seeds have the highest concentrations of cadmium, the main sources of dietary cadmium exposure (80%) in most Western populations are bread and other cereals, potatoes, root crops and vegetables due the comparatively high consumption of these products. In Asian populations, rice is the dominating source (Jarup and Åkesson, 2009). Mechanistically, cadmium may induce cancer in multiple ways. Possible non-oestrogen-mediated mechanisms of carcinogenicity are aberrant gene expression, oxidative stress, inhibition of DNA damage repair (Jin *et al*, 2003) and apoptosis (Joseph, 2009) or epigenetic alterations (Arita and Costa, 2009). Cadmium can also mimic the *in vivo* effects of oestrogen in reproductive tissues (Johnson *et al*, 2003). These oestrogen-mimicking effects may be mediated both via the oestrogen receptor (ER) (Byrne *et al*, 2009) and through alternative pathways in the absence of ERs (Filardo *et al*, 2008; Yu *et al*, 2010).

The aetiology of ovarian cancer is not fully understood. Results from experimental studies support a potential role of oestrogens to stimulate proliferation of ovarian surface epithelium cells (Syed *et al*, 2001; Spillman *et al*, 2010), but epidemiological evidence is more inconclusive. Whereas long-term use of any type of hormone replacement therapy seems to increase the risk of ovarian cancer (Lukanova and Kaaks, 2005), some studies observed a weaker association in users taking hormones containing oestrogen combined with progestin (Pearce *et al*, 2009). The observed favourable effect of progestins could explain these findings. Nevertheless, taken together, the results support the hypothesis of long-term elevated oestrogen concentrations as aetiologic important for this disease. Also in favour of the oestrogen hypothesis is the protective effect of oral contraceptive use, as it decreases ovarian oestrogen production (Lukanova and Kaaks, 2005). The oestrogen-mimicking properties of cadmium and the role of oestrogens in the aetiology of ovarian cancer may suggest a role of cadmium in this malignancy.

In a large population-based prospective cohort of Swedish women, we previously observed statistically significant positive association between dietary cadmium exposure and risk of endometrial cancer (Åkesson *et al*, 2008), a cancer form where oestrogen unopposed by progesterone is the main factor influencing risk (Akhmedkhanov *et al*, 2001). There are no studies on the association between cadmium exposure and ovarian cancer. This study aims to assess whether, in this same cohort, long-term dietary cadmium exposure is associated with the incidence of epithelial ovarian cancer.

## Materials and methods

### Study population

The Swedish Mammography Cohort was initiated in 1987–1990 when all women, born in 1914–48 and residing in Västmanland and Örebro counties of central Sweden, received a questionnaire including diet, lifestyle and reproductive factors, 74% responded (Åkesson *et al*, 2008). In 1997, data were updated; response rate was 70%. After exclusion of women with missing national registration number, implausible values for energy intake (mean ±3 s.d. value of log_e_-transformed energy intake), previous cancer diagnosis or bilateral oophorectomy, 60 889 women remained. The study was approved by the Regional Ethical Review Board in Stockholm, Sweden.

### Dietary assessment

Dietary intake was assessed by a 67- and 96-item food frequency questionnaire (FFQ) at baseline and in 1997, respectively, where the women were asked how often, on average, they had consumed each food item. The National Food Administration provided data on the food-cadmium content. We estimated the average daily cadmium exposure by multiplying the frequency of consumption by age-specific portion sizes and the average cadmium content in each food item (Åkesson *et al*, 2008). The validity of the baseline questionnaire was previously assessed by comparing FFQ  data with the average of four 1-week-weighted diet records among 129 randomly selected women of the cohort. Pearson correlation coefficients for the main cadmium-contributing food items ranged between 0.5–0.8.

### Identification of ovarian cancer cases and follow-up of the cohort

Incident cases of invasive epithelial ovarian cancer were identified by linkage of the cohort to the National Cancer Registry, which is close to 100% complete (Mattsson and Wallgren, 1984). Ascertainment of deaths was obtained from the Swedish Death Registry and oophorectomies, and hysterectomies were obtained from the National Hospital Discharge Registry.

### Statistical analysis

The estimated daily cadmium intake was adjusted for total energy intake of 1700 kcal (mean of the cohort) by using the residual method (Willett and Stampfer, 1986) and then categorized into tertiles. Follow-up was censored at the date of ovarian cancer diagnosis, death, bilateral oophorectomy or hysterectomy with unknown number of ovaries removed or 31 December 2009, whichever occurred first. Rate ratios (RR) and 95% confidence intervals (CIs) were estimated with Cox proportional hazards regression models with attained age as the timescale. In multivariable models, we adjusted for body mass index (BMI), post-secondary education, age at menarche, use of oral contraceptives, age at menopause, use of hormone replacement therapy, parity and age at first child birth. Linear trends across categories were tested using the median dietary cadmium intake values within categories as a continuous variable. All *P*-values were two-sided. Analyses on tumour subtypes were modelled with continuous cadmium exposure, due to the small number of cases. Statistical analyses were performed with STATA, version 11 (StataCorp., College Station, TX, USA).

## Results

The characteristics of the study population are presented in Table 1. Women in the highest tertile of cadmium intake were more likely to have a post-secondary education and to be never-smokers compared with those in the lowest tertile.

During a mean follow-up of 18.9 years (1 149 470 person-years) of 60 889 women, we identified 409 incident cases of epithelial ovarian cancer, including 215 serous, 27 mucinous, 62 endometrioid and 12 clear cell tumours. We found no association between dietary cadmium and risk of total epithelial ovarian cancer; neither after adjustment for age, nor in multivariable-adjusted analysis (Table 2). The lack of association remained also when starting follow-up in 1997 (multivariable-adjusted RR 1.03; 95% CI: 0.70–1.51 for all epithelial tumours) or in analysis comparing long-term consistently high intake of cadmium (> median) assessed twice (in 1987–1990 and 1997) (multivariable-adjusted RR 0.78; 95% CI: 0.55–1.10 for all epithelial tumours) with that of consistently low intake. Likewise, no association was observed for any subtype of ovarian cancer when modelled with continuous dietary cadmium exposure; multivariable RR for each 1 *μ*g per day increment of cadmium: 0.97 (95% CI: 0.93–1.02) for serous tumours, 0.94 (95% CI: 0.82–1.07) for mucinous tumours and 1.00 (95% CI: 0.92–1.08) for endometrioid and clear cell tumours.

Further, no association was present across strata of BMI, post-menopausal hormone use, oral contraceptive use or smoking, neither in age-adjusted (data not shown), nor in multivariable-adjusted models (Table 2).

## Discussion

In this prospective cohort of Swedish women, we observed no association between dietary cadmium exposure and the incidence of epithelial ovarian cancer. The lack of association persisted when starting follow-up later (1997), in the assessment of long-term consistent intake of cadmium and across strata of BMI, use of hormone replacement therapy, oral contraceptive use or smoking. To our knowledge, there are no other studies to date exploring the association between dietary cadmium exposure and ovarian cancer.

A possible reason for the lack of association in the present study, in contrary to that observed between dietary cadmium and endometrial cancer (Åkesson *et al*, 2008), is that oestrogen may not be the most important aetiologic factor for ovarian cancer (Risch, 1998). Indeed, a strong direct association was observed between circulating levels of oestrogen and endometrial cancer (Lukanova *et al*, 2004), but not ovarian cancer (Lukanova *et al*, 2003). Furthermore, even if obesity is associated with increased levels of circulating oestrogens, obesity seems to have a relatively weak adverse effect on ovarian cancer risk, if any (Renehan *et al*, 2010). Although cadmium may induce cancer also through non-oestrogen-mediated mechanisms (Jin *et al*, 2003; Arita and Costa, 2009; Joseph, 2009), the lack of an association in the present study does not support an important role of cadmium in the aetiology of ovarian cancer.

Our study has several strengths, including the prospective, population-based design, a relatively large number of cases and the nearly complete follow-up through linkage to population-based registers. We cannot exclude measurement error due to self-reported dietary intake, which could have influence on the lack of observed association. Additionally, due to a suggested ovarian cancer latency of 25–30 years (Risch, 1998), we may not have captured the relevant exposure time for ovarian carcinogenesis. In this study, we had the power (>80%) to detect increased relative risks of 1.4.

In conclusion, the present prospective study found no association between dietary cadmium exposure and risk of epithelial ovarian cancer. It suggests that dietary cadmium exposure is not likely to have a substantial role in ovarian cancer development.

## Figures and Tables

**Table 1:** Baseline (1987) age-standardized characteristics of 60 889 women by cadmium exposure

	**Tertiles of cadmium intake, μg day^−1^**		
	**1**	**2**	**3**
	**<14**	**14–16**	**>16**
Mean cadmium intake (μg per day)^a^	12	15	18
Mean age (years)	54	54	54
Mean body mass index (kg m^−2^)	25	25	25
Post-secondary education (%)	13	15	16
Age at menarche <13 years (%)	21	22	22
Oral contraceptive use (%)	44	44	44
Age at menopause ⩾51 years (%)	29	31	31
No use of post-menopausal hormones (%)^b^	41	43	43
			
*Number of children (%)*			
Nulliparous	11	10	11
⩾3	33	33	33
Age at first birth ⩾31 years (%)	14	15	15
			
*Smoking status (%)* c			
Never-smokers	31	36	37
Current smokers	16	14	13

a Dietary cadmium exposure was energy-adjusted by using the residual method.

b Based on 91% of the cohort at baseline with complete information from a supplemental questionnaire in 1987–1990.

c Based on women with complete information on smoking status from the 1997 questionnaire.

**Table 2:** Rate ratios (RR) and 95% confidence intervals (CI) of total epithelial ovarian cancer by tertiles of dietary cadmium intake (in 1987–1990) among 60 889 women of the Swedish Mammography Cohort and by subgroups of lifestyle and hormonal factors; follow-up 1987–2009

	**Tertiles of cadmium intake, μg day^−1^**			
	**1**	**2**	**3**	
**Total ovarian cancer**	**<14**	**14–16**	**>16**	***P*_trend_**
*Whole cohort*				
Person-years	385 538	384 795	379 137	
Cases, *n*	136	150	123	
Age-adjusted RR (95% CI)	1.00	1.08 (0.86–1.37)	0.88 (0.69–1.13)	0.31
Multivariable-adjusted RR (95% CI)^a^	1.00	1.09 (0.87–1.38)	0.89 (0.70–1.14)	0.34
				
*Subgroups*				
Normal weight (BMI 18.5–25 kg/m2)				
Cases, *n*	81	80	75	
Multivariable-adjusted RR (95% CI)^a^^,^^b^	1.00	1.01 (0.74–1.38)	0.99 (0.72–1.36)	0.95
Non-users of post-menopausal hormones				
Cases, *n*	57	65	55	
Multivariable-adjusted RR (95% CI)^a^	1.00	1.11 (0.78–1.58)	0.93 (0.64–1.35)	0.68
Non-users of oral contraceptives				
Cases, *n*	57	59	57	
Multivariable-adjusted RR (95% CI)^a^	1.00	0.95 (0.66–1.36)	0.87 (0.60–1.25)	0.46
Never smokers^c^				
Cases, *n*	30	29	33	
Multivariable-adjusted RR (95% CI)^a^	1.00	0.87 (0.52–1.45)	0.94 (0.57–1.55)	0.87

a Adjusted for attained age in years, BMI (18.5–24, 25–30, ⩾30 kg m^−2^), post-secondary education (⩾12, <12 years), age at menarche (<13, 13, >13 years), use of oral contraceptives (yes, no), age at menopause (⩽51, >51 years), use of post-menopausal hormones (yes, no), parity (nulliparous, 1–2, >2 children) and age at first birth (nulliparous, <26, 26–31, ⩾31years). Missing values were treated as a separate ‘missing category’ in the model.

b BMI was adjusted for as continuous.

c Based on 18 865 subjects with follow-up between 15 September 1997 and 31 December 2009.
